# Perforator-sparing basilar artery reconstruction normalizes pathological hemodynamics in vertebrobasilar dolichoectasia: a CFD study

**DOI:** 10.3389/fneur.2026.1790419

**Published:** 2026-04-10

**Authors:** Boqiang Cao, Zhuoyuan Li, Yidan Zhu, Shijie Na, Tao Liu, Haiping Ling, Tianzhu Xu, Yuhua Zhang, Xi Zhao, Zong Zhuang, Qingrong Zhang, Chunhua Hang

**Affiliations:** 1 Nanjing Drum Tower Hospital Clinical College of Nanjing Medical University, Nanjing, China; 2 Department of Neurosurgery, Sir Run Run Hospital, Nanjing Medical University, Nanjing, China; 3 Nanjing Drum Tower Hospital, The Affiliated Hospital of Nanjing University Medical School, Nanjing, China; 4 Central Research Institute, United Imaging Healthcare, Shanghai, China

**Keywords:** aneurysm-dissecting, computational fluid dynamics, flow diverter, hemodynamics, vertebrobasilar dolichoectasia

## Abstract

**Background:**

Vertebrobasilar dolichoectasia (VBD) carries high risks of stroke and hemorrhage, yet current treatments remain suboptimal and often compromise critical pontine perforators (PPs). We used computational fluid dynamics (CFD) to evaluate a novel hemodynamically-guided, perforator-sparing reconstruction strategy.

**Methods:**

Patient-specific CFD models from 24 VBD patients were compared with 24 age-sex-matched controls and virtual post-intervention models simulating idealized partial anterior basilar artery (BA) lumen reduction to establish hemodynamic optimization targets while preserving posterior wall PPs. Transient simulations quantified pressure, velocity, time-averaged wall shear stress (TAWSS), oscillatory shear index (OSI), and relative residence time (RRT) within the BA and nine modeled PPs per case.

**Results:**

Untreated VBD exhibited pathological hemodynamics: elevated aneurysmal pressure (101.73 ± 0.28 vs. 100.59 ± 0.87 mmHg, *p* < 0.001), flow stasis (velocity: 5.53 ± 3.52 vs. 12.41 ± 3.44 cm/s, *p* < 0.001), reduced TAWSS (0.007 ± 0.004 vs. 0.03 ± 0.01 Pa, *p* < 0.001), and increased OSI (0.07 ± 0.04 vs. 0.01 ± 0.00) and RRT (12.73 ± 13.81 vs. 0.33 ± 0.11 /Pa, all *p* < 0.001). Perforator hemodynamics remained comparable to controls. Virtual reconstruction of BA normalized all aneurysmal parameters toward physiological levels (Post vs. Pre: all *p* < 0.001) without adversely affecting hemodynamics in the modeled PPs under the assumptions of the CFD framework. Strong correlations existed between BA volume and adverse hemodynamics, with therapeutic benefits approaching saturation at about 250 mm^3^.

**Conclusion:**

This in silico study demonstrates that perforator-sparing partial reconstruction can normalize VBD’s pathological hemodynamics without adversely affecting hemodynamics in the modeled PPs, and establishes a quantitative framework for future endovascular device development, though clinical translation requires validation through experimental models and prospective trials.

## 1 Introduction

Vertebrobasilar dolichoectasia (VBD) is a cerebrovascular disorder characterized by pathological elongation, tortuosity, and dilation of the vertebrobasilar arteries. Its natural history is often unfavorable, leading to a high lifelong risk of severe stroke, brainstem compression, and hemorrhage (1, 2). Consequently, clinical management remains challenging (3–5). Current treatments, medical, surgical or endovascular, are suboptimal. Medical therapy is palliative and fails to address the structural pathology, while surgical and endovascular procedures carry significant risks, including the occlusion of critical pontine perforators (PPs) and inconsistent outcomes (6–8). This highlights the lack of a treatment strategy directly guided by the reversal of VBD’s hemodynamic disturbances, such as flow stasis and aberrant wall shear stress (WSS) (9–12), that contribute to thrombosis and vascular injury.

To address this issue, we proposed and computationally evaluated a hemodynamically-guided treatment strategy for VBD. Rather than simulating any specific existing device, this study employs an idealized geometric reconstruction approach to establish the hemodynamic endpoints that any effective treatment should achieve. This strategy aims to define quantitative benchmarks—such as optimal volume reduction thresholds and perforator-preserving criteria—against which future device-specific simulations and clinical interventions can be evaluated.

The core of this virtual treatment concept focuses on two principles: reducing the aneurysmal volume of the basilar artery (BA) aneurysm and achieving perforator-sparing reconstruction to minimize interference with PPs. Using patient-specific computational fluid dynamics (CFD) models, we compared three cohorts: healthy controls (Control group), untreated VBD patients (Pre group), and patients after virtual intervention (Post group). The objectives of this study were: (1) to characterize the pathological hemodynamic features of VBD by comparing it with healthy controls; (2) to design a conceptual BA reconstruction strategy that aims to correct adverse flow in the aneurysm while preserving PP perfusion; and (3) to quantitatively evaluate the potential of this strategy in normalizing pathological flow patterns and key hemodynamic parameters.

This study thus introduces and validates a novel treatment concept for VBD, grounded in the correction of its pathogenic hemodynamics. The results highlight the feasibility and hemodynamic benefits of a virtual, perforator-sparing BA reconstruction, offering a principled and promising direction for overcoming the limitations of current management strategies.

## 2 Materials and methods

### 2.1 Study cohorts

In this study, patients with VBD were retrospectively identified from our institutional imaging database from August 2018 to August 2024. The diagnosis was based on radiographic criteria previously described by Flemming et al. (13), defined as dilation of the vertebrobasilar artery to greater than 1.5 times the normal diameter without a clearly definable neck and with visible involvement of the basilar trunk artery, as observed on magnetic resonance angiography (MRA), computed tomography angiography (CTA), or digital subtraction angiography (DSA).

The initial screening yielded 43 patients meeting the VBD imaging criteria. Two senior neurosurgeons and one neuroradiology specialist independently reviewed all images. Exclusion criteria comprised: (1) suboptimal image quality; (2) significant stenosis (>50%) or occlusion in the posterior circulation; and (3) prior posterior circulation aneurysm clipping or stenting. Ultimately, 24 patients were enrolled in the VBD cohort. The control group consisted of 24 subjects with normal BA morphology on MRA, individually matched by age and sex.

### 2.2 Vascular and perforator modeling

**Figure 1:**
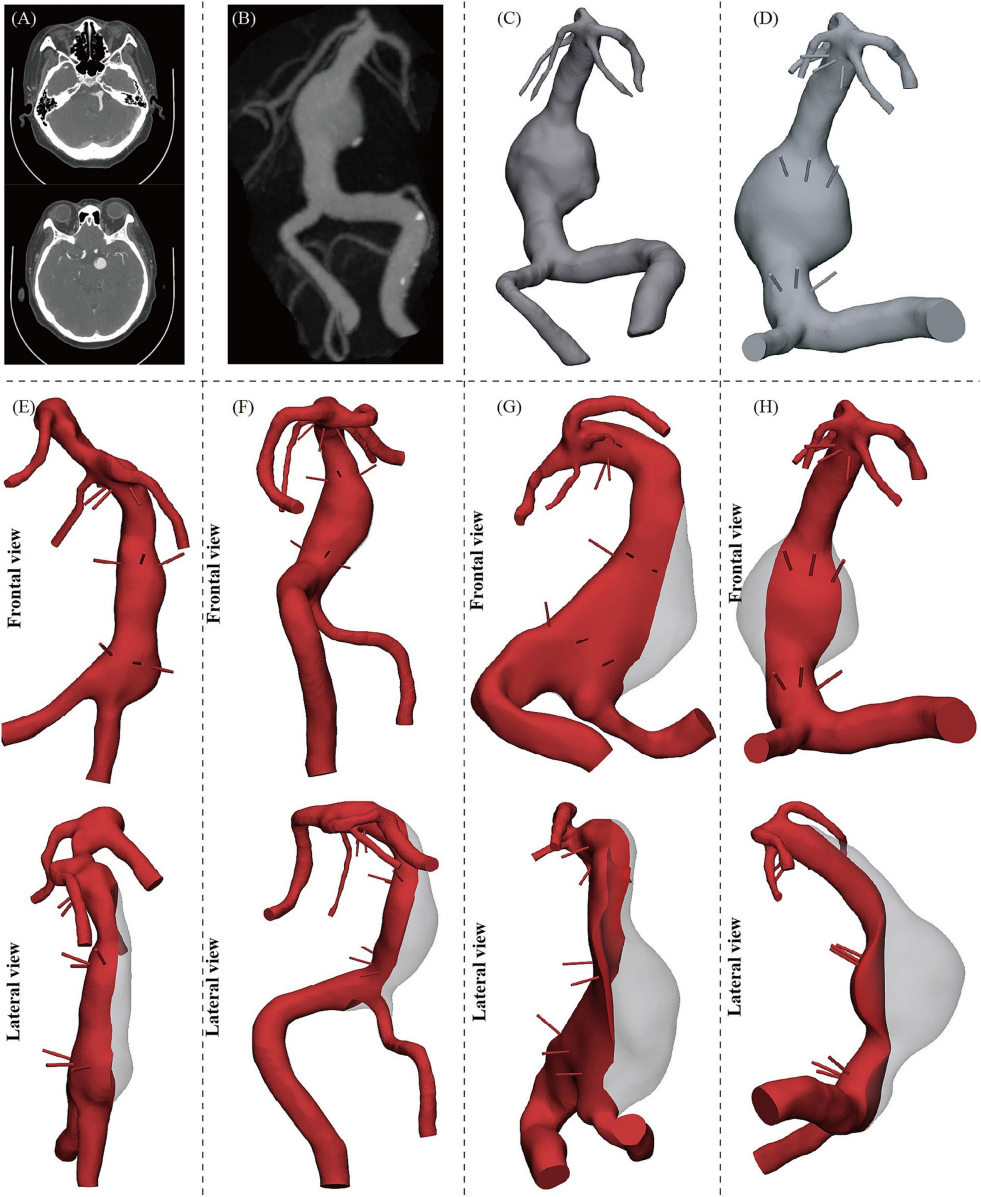
Process of vascular model construction. **(A)** Original imaging data of the patient (CTA shown as an example). **(B)** Three-dimensional reconstruction of the original imaging data. **(C)** 3D model extraction. **(D)** Further design and construction of PPs on the model. **(E–H)** Reconstructed models of several typical VBD cases in frontal and lateral views, with the red color indicating the post-reconstruction morphology.

Three-dimensional reconstructions of the BA, bilateral VAs, PCAs, and SCAs were performed from patient-specific imaging data of the Pre group using 3D Slicer 5.8.1 and Geomagic Wrap 2021, with one model excluding PCAs due to a fetal-type variant. The process from imaging data to 3D reconstruction is detailed in Figures 1A–D. Although PPs are often not clearly visualized and may be absent in VBD (14), we incorporated them into all models via a standardized design approach. This involved virtually dividing the BA into superior, middle, and inferior thirds, and manually modeling three PPs (left, midline, right) on the posterior wall of each segment as perpendicular cylindrical conduits [0.5 mm diameter (15), 5 mm length], as shown in Figures 1E–H. Vascular models for the Control group were generated using the same pipeline.

### 2.3 Virtual hemodynamic optimization modeling

The objective of this study was to establish hemodynamic optimization targets rather than to simulate specific endovascular devices. Therefore, we employed an idealized geometric modification approach to represent the theoretical upper limit of hemodynamic improvement achievable through lumen reduction.

Post-treatment models were created by geometrically subtracting the anterior portion of the dilated BA lumen while meticulously preserving the posterior wall and perforator origins. This approach intentionally simplifies the complex geometry of actual endovascular implants (e.g., FDs) to isolate the pure hemodynamic effect of volume reduction from device-specific factors such as mesh porosity, strut thickness, and wall apposition.

### 2.4 Definition of boundary conditions

CFD simulations were performed under transient conditions to resolve the time-dependent hemodynamic variations within a cardiac cycle. As this is a retrospective study lacking patient-specific *in vivo* flow data, a standardized set of physiological boundary conditions was applied uniformly to all models (Control, Pre, and Post groups). This approach ensures that any observed hemodynamic differences can be attributed primarily to geometric variations rather than discrepancies in inflow or outflow conditions.

These boundary conditions were acquired from a typical VBD patient (not included in the study cohort). A time-varying volumetric flow rate waveform was prescribed at the inlet (VA confluence), while all outlets (PCAs, SCAs and PPs) were assigned individual time-varying pressure waveforms.

### 2.5 CFD setup and execution

Transient CFD simulations were performed using the finite-volume solver ANSYS CFX (v.19.2). Blood was modeled as an incompressible Newtonian fluid (density: 1050 kg/m^3^; viscosity: 0.0035 Pa·s), and vessel walls were treated as rigid with no-slip conditions.

The vascular geometry of each model was discretized into an unstructured computational mesh using ANSYS ICEM CFD (v.19.2). To ensure accurate resolution of flow, especially near the vessel wall, a five-layer prismatic boundary layer was applied, and a mesh independence study confirmed that key results were not affected by further refinement.

The simulations employed a time step of 0.005 s to resolve the cardiac cycle. Three consecutive cycles were computed to eliminate initial transients, with results from the final cycle used for hemodynamic analysis.

### 2.6 Hemodynamic parameter extraction and analysis

Hemodynamic analysis focused on the BA lumen and the modeled PPs. For the BA, we extracted globally representative scalar values for wall pressure, time-averaged wall shear stress (TAWSS), oscillatory shear index (OSI), and relative residence time (RRT) by first calculating a surface-area-weighted average for each time point, then averaging over the cardiac cycle. Bulk flow was similarly assessed by time-averaging the volumetric flow rate across four transverse planes.

For the PPs, the pressure at the vessel wall and the time-averaged flow rate at the outlet were computed for each of the nine artificially modeled vessels per case. For statistical analysis, each of the nine PPs within a given model was treated as an independent observation. We acknowledge that this approach has limitations as the nine PPs per patient share identical boundary conditions and originate from the same parent vessel, which may inflate statistical power and overestimate significance. Therefore, perforator-related statistical findings should be interpreted descriptively rather than as fully independent observations, and our conclusions regarding perforator hemodynamics are constrained by the standardized modeling approach.

### 2.7 Pressure normalization

To enable a meaningful comparison of hemodynamic parameters across groups and to facilitate a physiologically relevant interpretation of the results, the simulated pressure data underwent a normalization procedure. In reality, proximal perfusion pressure is relatively consistent among individuals. However, in our simulations, applying uniform outflow boundary conditions, though it may be the most feasible solution, led to varying computed inlet pressures across models, which does not reflect this physiological reality.

We therefore adjusted the absolute pressure field of each model by a constant offset, such that the time-averaged pressure at the vertebral artery confluence equaled a common reference value. The normalization was performed using the following formula:


P_norm=P_orig+(P_inlet_ref−P_inlet_sim)

where *P_norm* is the normalized pressure, *P_orig* is the pressure obtained directly from the simulation, *P_inlet_ref* is the boundary condition time-averaged inlet pressure, and *P_inlet_sim* is the simulated time-averaged inlet pressur.

This process preserved the intrinsic pressure gradients and flow resistance within each model while standardizing the inlet perfusion pressure. All reported pressure data are these normalized values.

In contrast, wall shear stress-derived parameters (TAWSS, OSI, RRT) were not normalized. These parameters are primarily determined by flow velocity gradients, which were directly driven by a uniform inlet flow waveform applied to all models.

It is important to emphasize that these normalized pressure values represent relative measures intended for comparative analysis across groups within the computational model, and do not represent absolute physiological pressure differences *in vivo*. The small absolute differences observed between groups, while statistically significant, should be interpreted within the context of this normalization framework rather than as clinically meaningful absolute pressure changes.

### 2.8 Statistical analysis

All analyses were conducted using Python 3.12, with the *scipy.stats* and *statsmodels* libraries. Continuous variables are presented as mean ± standard deviation. Normality (Shapiro–Wilk test) and homogeneity of variances (Levene’s test) were assessed for all parameters. Inter-group comparisons across the three independent groups (Control, Pre, Post) were conducted using one-way ANOVA (or the Kruskal-Wallis test if parametric assumptions were violated), with Tukey’s HSD test (or Dunn’s test with Bonferroni correction) applied for post-hoc pairwise comparisons. To specifically evaluate the effect of the simulated intervention, hemodynamic parameters between the Pre and Post groups were compared using paired t-tests (or the Wilcoxon signed-rank test for non-normally distributed differences). Furthermore, the relationship between hemodynamic parameters and vascular morphology was examined by calculating Pearson (or Spearman) correlation coefficients between these parameters and the corresponding BA volume across all groups. Outliers were identified using the interquartile range (IQR) method (values below Q1–1.5 × IQR or above Q3 + 1.5 × IQR). A sensitivity analysis confirmed that removing only the single most extreme observation per group yielded results consistent with full IQR-based exclusion; this conservative approach was adopted to retain maximum statistical power. The impact of this exclusion is illustrated using comparative violin and box plots in the Supplementary materials. Statistical significance for all tests was defined as *p* < 0.05.

## 3 Results

Prior to analysis, statistical outliers were identified and excluded. One outlier (4.2% of the group) was removed from the Pre and Post groups for each of the following parameters: Aneurysm Pressure, Velocity, and TAWSS, as well as PPs Pressure. The distributions of all data before (Supplementary Image 1) and after (Supplementary Image 2) this exclusion are comparatively visualized.

### 3.1 Study population and baseline characteristics

Morphologically, the Pre group exhibited a dramatically enlarged and more tortuous BA across all measured parameters (volume, length, radius, and cross-sectional area) compared to the Control group, as detailed in Supplementary Table 1. The simulated endoluminal reconstruction successfully reduced the BA volume, radius, and cross-sectional area in the Post group, achieving values intermediate between the Control and Pre group.

### 3.2 Pathological hemodynamic alterations in VBD

Hemodynamic alterations in VBD patients primarily concentrated within the dilated BA segment, with adjacent PPs largely unaffected.

**Figure 2:**
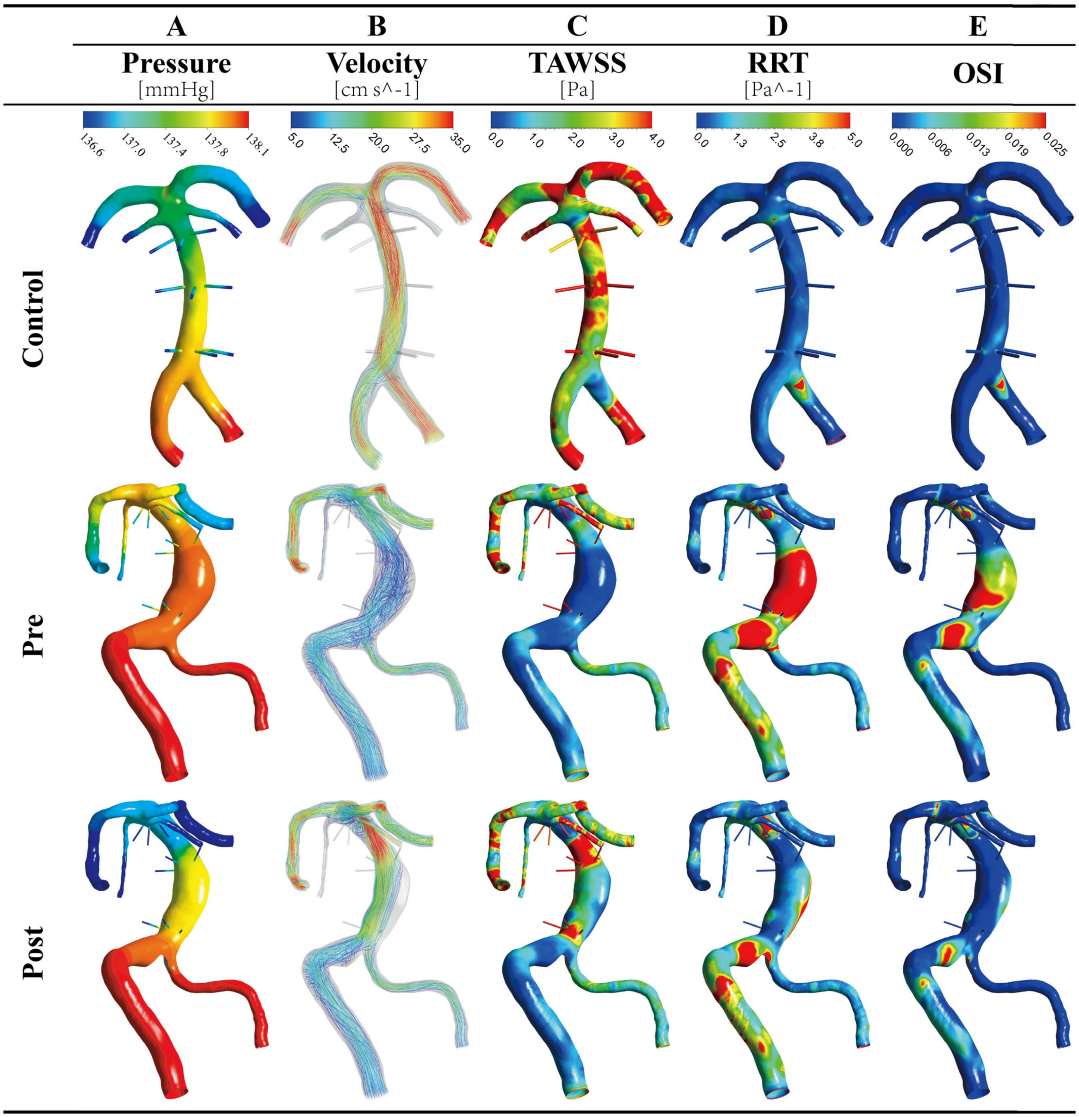
Hemodynamic changes following simulated partial remodeling. Representative results from a healthy control (Control), an untreated VBD case (Pre), and the same case after virtual intervention (Post) are shown. The intervention, which reduced the ventral lumen, redirected flow dorsally. This reversed the adverse hemodynamic state seen in the pre group—characterized by elevated pressure, flow stasis, low TAWSS, high RRT, and OSI—and restored parameters toward a more physiological state in the post group.

Within the aneurysmal sac, a distinctly pathological flow environment was observed: a marked elevation in wall pressure both inside the dilated sac and at distal BA branches (Figure 2A; Table 1) was coupled with substantially reduced flow velocity (Figure 2B; Table 1) and complex, unstable vortices—most pronounced at the region of maximal BA dilation (Figure 2B; Supplementary Video 1). Furthermore, WSS analysis, particularly within the dilated segment, revealed a flow profile conducive to atherogenesis and thrombosis, characterized by significantly lower TAWSS and elevated OSI and RRT compared to healthy controls (Figures 2C–E; Table 1).

**Table 1:** Statistical comparisons of hemodynamic parameters in the aneurysm and PPs.

Parameter	C^*^	Pre	Post	P_overall_	P_pairwise_			P_paired comparison_
					C-Pre	Pre-Post	C-Post	Pre-Post
Pressure (mm Hg)								
Aneurysm	100.59 ± 0.87	101.73 ± 0.28	100.51 ± 1.63	**<0.001**	**<0.001**	**<0.001**	0.875	**<0.001**
PP	99.15 ± 1.60	99.63 ± 1.89	98.10 ± 3.02	**0.015**	0.272	**0.021**	0.372	**<0.001**
Velocity (cm/s)								
Inner Aneurysm	12.41 ± 3.44	5.53 ± 3.52	10.16 ± 7.46	**<0.001**	**<0.001**	**<0.001**	**<0.001**	**<0.001**
Inner PP	9.53 ± 5.86	11.48 ± 8.48	13.96 ± 12.01	0.093	1.000	0.458	0.094	**0.006**
TAWSS (Pa)								
Aneurysm	0.03 ± 0.01	0.007 ± 0.004	0.028 ± 0.025	**<0.001**	**<0.001**	**<0.001**	**0.0018**	**<0.001**
OSI								
Aneurysm	0.01 ± 0.00	0.07 ± 0.04	0.02 ± 0.01	**<0.001**	**<0.001**	**<0.001**	**<0.001**	**<0.001**
RRT (/Pa)								
Aneurysm	0.33 ± 0.11	12.73 ± 13.81	2.56 ± 1.73	**<0.001**	**<0.001**	**<0.001**	**<0.001**	**<0.001**

The bolded values indicate significant differences. P_overall_ is the p-value of the ANOVA for three sets of data. P_pairwise_ is the p-value of the post hoc test for two sets of data. P_paired comparison_ is the p-value of the paired test for Pre and Post. Non-parametric tests were used when the relevant assumptions were not satisfied, as detailed in the Statistical Analysis section. ^*^C represents Control, the healthy controls.

However, hemodynamic parameters in the PPs—including both pressure and flow velocity—showed no statistically significant differences between VBD patients and healthy controls. This localized pattern underscores that the dominant hemodynamic pathology originates from the aneurysmal dilation itself (Figure 2; Table 1).

It is important to note that all pressure values reported are normalized relative measures (see Methods 2.7), and the small absolute differences between groups, while statistically significant, should not be interpreted as representing clinically meaningful absolute pressure gradients *in vivo*.

### 3.3 Hemodynamic improvements following the partial remodeling strategy

The simulated partial remodeling strategy effectively reversed the pathological hemodynamics within the BA aneurysm while preserving stability in the PPs. Following the intervention, the virtual treated Post group exhibited a significant reduction in aneurysmal wall pressure and a marked increase in flow velocity compared to the Pre state, alongside the disappearance of unstable vortices (Figures 2A,B; Supplementary Video 1). Concomitantly, WSS metrics shifted toward a more stable, physiological profile, with increased TAWSS and decreased OSI and RRT, closely resembling the pattern observed in healthy controls (Figures 2C–E).

Importantly, while minor changes in pressure and flow were detected in the PPs between the Pre and Post states, all PP hemodynamic parameters in both the Pre and Post groups remained statistically indistinguishable from those in healthy controls. This confirms that the primary hemodynamic pathology was localized to the aneurysm and that, within the limitations of our standardized PP modeling approach, the remodeling strategy achieved its therapeutic aim without adversely affecting hemodynamics in the modeled PPs.

### 3.4 Correlation between BA volume and hemodynamic parameters

**Figure 3:**
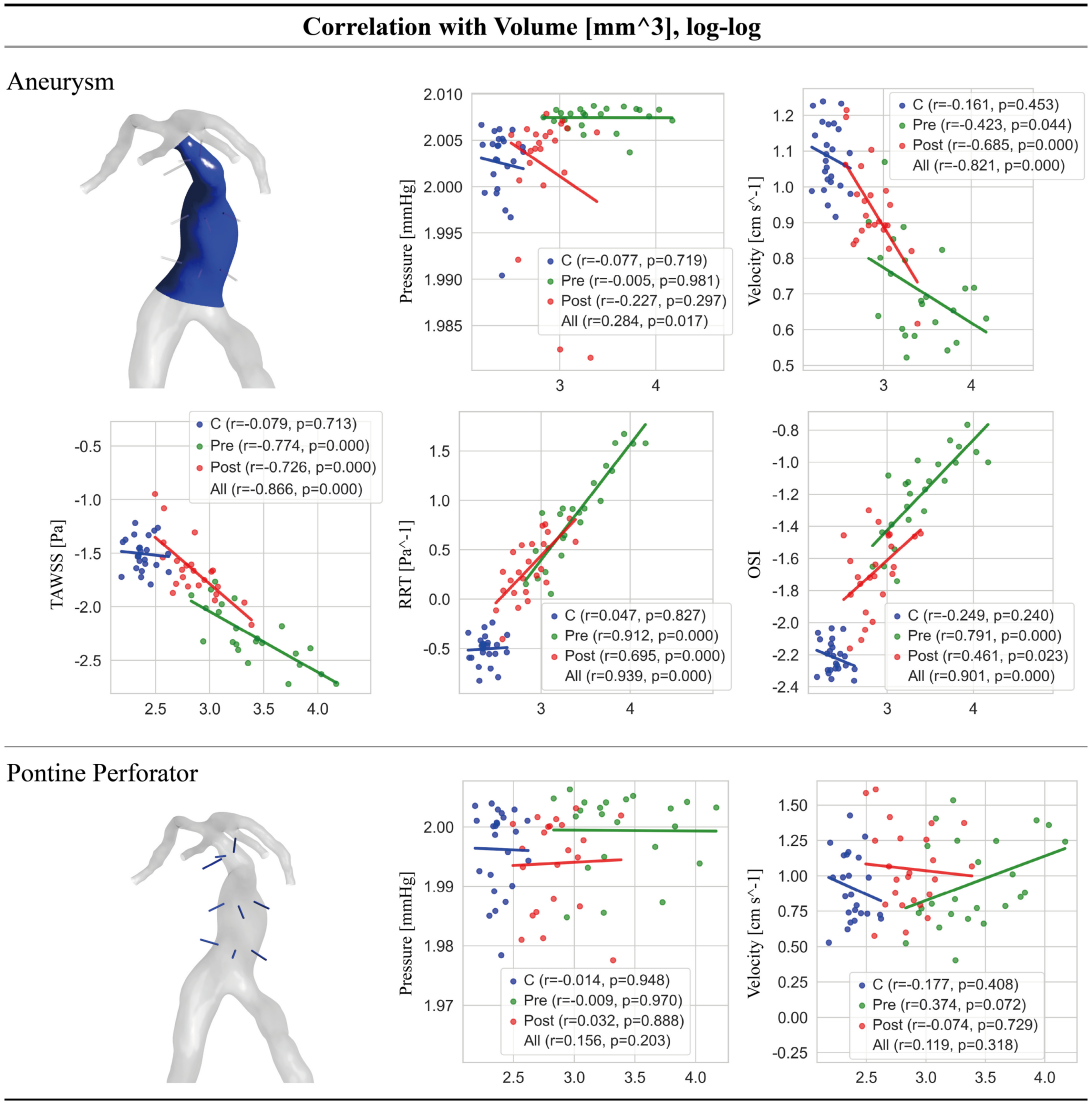
Correlations between BA volume and hemodynamic parameters.

Analysis of the relationship between BA volume and hemodynamic parameters revealed distinct patterns across groups (Figure 3).

In untreated Pre group, larger BA volume was strongly associated with adverse intra-aneurysmal hemodynamics: it correlated negatively with flow velocity (*r* = −0.423, *p* = 0.044) and TAWSS (*r* = −0.774, *p* < 0.001), and positively with OSI (*r* = 0.791, *p* < 0.001) and RRT (*r* = 0.912, *p* < 0.001). In contrast, no significant correlations were found between BA volume and any hemodynamic parameter in the PPs.

Following the virtual intervention (the Post group), the strength of these correlations with adverse shear metrics weakened (OSI: *r* = 0.461, *p* = 0.023; RRT: *r* = 0.695, *p* < 0.001), while the negative correlation with velocity became stronger (*r* = −0.685, *p* < 0.001). Notably, hemodynamics in the PPs remained uncorrelated with BA volume in the Post group, as observed in the Pre group.

When data from all three groups were pooled, highly significant correlations (all *p* < 0.001) were confirmed across the spectrum of BA volumes: larger volume was associated with lower intra-aneurysmal velocity (*r* = −0.821) and TAWSS (*r* = −0.866), and higher OSI (*r* = 0.901) and RRT (*r* = 0.939). A significant but weak negative correlation was also found between BA volume and intra-aneurysmal pressure in the pooled analysis (*r* = −0.227, *p* = 0.017). In the healthy control group, BA volume was not significantly correlated with any of the measured hemodynamic parameters.

Scatter plots illustrate correlations between BA volume and key hemodynamic parameters in the aneurysm sac and PPs across Control (C, blue), Pre-intervention (Pre, red), and Post-intervention (Post, green) groups. In the Pre state, larger BA volume was strongly correlated with adverse intra-aneurysmal hemodynamics (velocity, TAWSS, RRT and OSI). The virtual remodeling strategy markedly mitigated these pathological impacts. In contrast, BA volume showed no significant correlation with hemodynamic parameters (pressure, velocity) in the PPs across all groups, indicating that perfusion to these critical branches was preserved regardless of the aneurysmal state or intervention.

## 4 Discussion

This CFD study revealed distinct hemodynamic challenges in VBD, validating a hemodynamically-guided, perforator-protecting treatment strategy. By virtually reducing the aneurysm volume, we demonstrated a reversal of the pathological flow environment while preserving the hemodynamic integrity of critical perforating arteries. These findings translate the abstract principles of VBD pathogenesis into a concrete, quantifiable therapeutic target, offering potential guidance for future clinical interventions.

### 4.1 Hemodynamic pathogenesis of VBD complications

The hemodynamic characteristics of VBD are closely related to its complications. Elevated wall pressure provides a mechanical basis for progressive aneurysmal expansion and the risk of hemorrhage (16). Reduced TAWSS is associated with mechanisms such as intimal damage (17, 18) and aneurysm rupture (19). Elevated OSI reflects an increased oscillatory nature of flow direction, which correlates with intimal disfunction and aneurysm rupture (18). High RRT, representing prolonged blood residence time, is implicated in endothelial cell activation and promotes thrombus formation within the aneurysm (20). High RRT, together with low flow velocity and unstable vortices, characterizes a sluggish and chaotic flow pattern. This combination aligns with the classic Virchow’s triad: stasis, endothelial injury, and a consequent tendency toward hypercoagulability (21). This mechanistic insight explains the high thromboembolic risk in VBD, where in-situ thrombosis within the aneurysm sac is a primary source of distal emboli (22).

Notably, our results indicate that PPs are hemodynamically shielded from the adverse effects of the adjacent aneurysm, with their patency remaining comparable to healthy controls. This suggests that, within the context of our modeled perforators with standardized geometry and boundary conditions, brainstem ischemia in VBD is more likely attributable to structural mechanisms, such as intra-aneurysmal thrombosis, atherosclerosis, dissection, or mechanical stretching and stenosis of the PPs (22, 23), rather than hemodynamic reasons alone.

### 4.2 Therapeutic rationale and quantifiable target of BA reconstruction

This study demonstrates that the BA reconstruction strategy, by geometrically reducing the anterior lumen, reversed key adverse hemodynamic parameters without adversely affecting hemodynamics in the modeled PPs. Post-intervention hemodynamics were characterized by decreased wall pressure, increased flow velocity, attenuated vortex complexity, elevated TAWSS, and reduced OSI and RRT.

Each of these changes carries significant clinical implications supported by prior research. In the context of our normalized pressure framework, decreased wall pressure relative to the pre-intervention state may be associated with reduced aneurysm rupture risk (16), though the clinical significance of these relative changes requires further validation. Increased flow velocity and attenuated vortex complexity can lower the risk of intra-aneurysmal thrombosis (21). Low WSS is a well-established marker of aneurysm instability; for instance, the proportion of low WSS area is significantly higher in the dome of ruptured versus unruptured intracranial aneurysms (24). Low WSS is further associated with aneurysm wall enhancement (25), increased wall permeability (26), and endothelial dysfunction (27), potentially promoting inflammation, endothelial injury, and thrombosis to accelerate aneurysm progression. High OSI is closely linked to aneurysm instability and rupture, commonly observed in ruptured cases (28, 29), and correlates with wall enhancement (30) and co-localizes with low WSS regions (31), suggesting its role in endothelial dysfunction and structural degradation. Elevated RRT is also prevalent in ruptured aneurysms (28, 29, 32). Notably, the triad of low TAWSS, high OSI, and high RRT has been consistently validated in association with ruptured or growing aneurysms (11, 24, 29, 32). This hemodynamic profile is also implicated in thrombosis within abdominal aortic aneurysms (33) and other non-aneurysmal cerebrovascular diseases (34). Collectively, these hemodynamic parameters influence endothelial function, wall integrity, and thrombotic propensity.

In our model, the BA reconstruction strategy shifted these parameters toward a more physiological state. This transition from a pro-thrombotic to a normalized flow profile provides a hemodynamic rationale for the intervention to mitigate both thromboembolic and hemorrhagic risks.

Furthermore, our correlation analysis established a quantifiable therapeutic target. A dose-dependent relationship was identified between the residual BA volume and the improvement in hemodynamic metrics. The beneficial effects approached saturation when the BA lumen geometry was normalized to approximately 250 mm^3^, suggesting this as a potential optimal anatomical endpoint for the intervention.

### 4.3 Clinical translation and strategy in a pragmatic reality

Though the central contribution of this study lies in introducing a hemodynamically-guided, “target-first” strategy for VBD treatment—defining the hemodynamic and anatomical endpoints (e.g., optimal volume reduction and perforator preservation) that an ideal treatment should achieve—its implementation remains conceptual. The current model relies on an idealized geometric reconstruction. Future work must therefore translate this target into practice by simulating actual FD devices (e.g., tubular mesh structures with specific porosity) to assess how closely their hemodynamic performance approximates the benchmarks established herein. Discrepancies between idealized and device-specific simulations will provide critical feedback for optimizing next-generation implant designs tailored to the unique challenges of VBD anatomy.

In current clinical practice, BA reconstruction is typically attempted using low-profile braided stents or FDs (35) Our findings reinforce the principle that lumen reconstruction should primarily target the anterior BA wall to spare posteriorly originating PPs. However, stent deployment itself entails significant risks: coverage of PP ostia may impede branch perfusion (36–38), while stent malposition or its inherent flow-altering structure may promote in-stent thrombosis and further compromise PP patency (39).

Consequently, proactive peri-procedural thrombus management may be important for success (40). This encompasses optimized antiplatelet or anticoagulant regimens and meticulous technique to ensure complete stent-wall apposition. Although seldom reported, pretreatment intraluminal thrombus clearance prior to stent deployment could offer additional benefits—preventing thrombotic debris from embolizing into PP ostia and facilitating smoother stent expansion. Moreover, adjunctive coiling within the anterior aneurysmal segment may enhance stent stability and improve the geometry of the reconstructed lumen, representing a feasible coiling-assisted posterior wall stenting strategy. Collectively, these technical considerations underscore that the hemodynamic benefits demonstrated in our virtual model must be pursued alongside careful mitigation of device-related risks in clinical translation.

### 4.4 Limitations

This proof-of-concept computational simulation study has inherent limitations. The simulations assumed rigid vessel walls, neglecting fluid–structure interaction and potential arterial compliance. The virtual intervention employed planar cutting to isolate the hemodynamic effect of volumetric reduction, thereby establishing theoretical optimization targets rather than simulating specific device architectures; consequently, translating these targets into clinical predictions requires subsequent device-specific CFD analyses and experimental validation. Furthermore, the model utilized standardized major PP representations with identical geometry (0.5 mm diameter, 5 mm length, perpendicular orientation) and treated each of the nine PPs per patient as independent observations in statistical analysis. This approach does not account for the known anatomical heterogeneity, variable presence, caliber differences, or autoregulatory behavior of PPs *in vivo*, particularly in VBD where PPs may be absent or anatomically distorted (14). The statistical treatment of PPs as independent observations may artificially increase statistical power. Consequently, our findings regarding PP preservation should be interpreted as demonstrating that the virtual intervention did not adversely affect hemodynamics in modeled PPs under the specific assumptions of our CFD framework, rather than as definitive evidence of clinical PP safety. Finally, the clinical efficacy and safety of this approach must be validated through future experimental models and carefully designed clinical trials.

## 5 Conclusion

This CFD study demonstrates that a perforator-sparing, partial volumetric reduction strategy can effectively normalize the pathological hemodynamics within VBD aneurysms. The intervention directly addresses the stasis and endothelial injury implicated in thrombosis and progression, while our correlation analysis defines a quantifiable, physiologically-grounded anatomical target for therapy. This work provides a foundational hemodynamic rationale to guide future development and refinement of clinical interventional strategies that move beyond simple occlusion or diversion, toward the goal of tailored, hemodynamically-guided BA reconstruction for this complex and high-risk disease.

## Data Availability

The raw data supporting the conclusions of this article will be made available by the authors, without undue reservation.

## References

[1] Smoker WR Price MJ Keyes WD Corbett JJ Gentry LR. High-resolution computed tomography of the basilar artery: 1. Normal size and position. AJNR Am J Neuroradiol. (1986) 7:55–60. 3082144 PMC8334772

[2] Pico F Labreuche J Amarenco P. Pathophysiology, presentation, prognosis, and management of intracranial arterial dolichoectasia. Lancet Neurol. (2015) 14:833–45. doi: 10.1016/S1474-4422(15)00089-7, 26194931

[3] Xu DS Levitt MR Kalani MYS Rangel-Castilla L Mulholland CB Abecassis IJ . Dolichoectatic aneurysms of the vertebrobasilar system: clinical and radiographic factors that predict poor outcomes. J Neurosurg. (2018) 128:560–6. doi: 10.3171/2016.10.JNS161041, 28387624

[4] Bin-Alamer O Qedair J Palmisciano P Mallela AN Nayar GM Lu VM . Dolichoectatic vertebrobasilar aneurysms: a systematic review and meta-analysis of management strategies and outcomes. Neurosurg Focus. (2023) 54:E9. doi: 10.3171/2023.2.FOCUS22650, 37127027

[5] Rezai Jahromi B Dashti R Ota N Dabbagh Ohadi MA Srinivasan V Fiorella D . Natural history of dolichoectatic vertebrobasilar aneurysms: a multinational study. J Neurosurg. (2024) 142:1–11. doi: 10.3171/2024.7.JNS232341 39705689

[6] Lawton MT Abla AA Rutledge WC Benet A Zador Z Rayz VL . Bypass surgery for the treatment of Dolichoectatic basilar trunk aneurysms: A work in Progress. Neurosurgery. (2016) 79:83–99. doi: 10.1227/NEU.0000000000001175, 26671632 PMC4956413

[7] Bhogal P Pérez MA Ganslandt O Bäzner H Henkes H Fischer S. Treatment of posterior circulation non-saccular aneurysms with flow diverters: a single-center experience and review of 56 patients. J NeuroInterventional Surg. (2016) 9:471–81. doi: 10.1136/neurintsurg-2016-012781, 27836994 PMC5520279

[8] Griessenauer CJ Ogilvy CS Adeeb N Dmytriw AA Foreman PM Shallwani H . Pipeline embolization of posterior circulation aneurysms: a multicenter study of 131 aneurysms. J Neurosurg. (2019) 130:923–35. doi: 10.3171/2017.9.JNS171376, 29726768

[9] Amin-Hanjani S Du X Rose-Finnell L Pandey DK Richardson D Thulborn KR . Hemodynamic features of symptomatic vertebrobasilar disease. Stroke. (2015) 46:1850–6. doi: 10.1161/STROKEAHA.115.009215, 25977279 PMC4607265

[10] Brinjikji W Chung B Yong-Hong D Wald JT Mut F Kadirvel R . Hemodynamic characteristics of stable and unstable vertebrobasilar dolichoectatic and fusiform aneurysms. J Neurointerv Surg. (2018) 10:1102–7. doi: 10.1136/neurintsurg-2018-013756, 29549120

[11] Jiang Y Ge L Lu G Wan H Chen Q Zou R . Wall enhancement predictive of abnormal hemodynamics and ischemia in vertebrobasilar non-saccular aneurysms: a pilot study. Front Neurol. (2023) 14:1108904. doi: 10.3389/fneur.2023.1108904, 37333010 PMC10272805

[12] Liu Q Nie X Vergouwen MDI Wang Y He H Wu J . Gadolinium-enhanced Aneurysm Wall imaging and risk of intracranial aneurysm growth or rupture. JAMA Neurol. (2025) 82:1135–43. doi: 10.1001/jamaneurol.2025.3209, 40920406 PMC12418222

[13] Flemming KD Wiebers DO Brown RD Link MJ Nakatomi H Huston J . Prospective risk of hemorrhage in patients with vertebrobasilar nonsaccular intracranial aneurysm. J Neurosurg. (2004) 101:82–7. doi: 10.3171/jns.2004.101.1.0082, 15255255

[14] Dobrocky T Piechowiak EI Goldberg J Barvulsky Aleman E Nicholson P Lynch J . Absence of pontine perforators in vertebrobasilar dolichoectasia on ultra-high resolution cone-beam computed tomography. J Neurointerv Surg. (2020) 13:580–4. doi: 10.1136/neurintsurg-2020-016818, 33087525 PMC8142461

[15] Tomaszewski M Kucewicz M Rzepliński R Małachowski J Ciszek B. Numerical aspects of modeling flow through the cerebral artery system with multiple small perforators. Biocybern Biomed Eng. (2024) 44:341–57. doi: 10.1016/j.bbe.2024.04.002

[16] Morales-Verdugo J Pérez-Rojas F Figueroa-Figueroa A Lagos-Fica J Vera-Paredes J García-Suárez O . Detection, cerebrovascular complications and risk factors associated with vertebrobasilar dolichoectasia: a scoping review. Front Neurol. (2025) 16:1668912. doi: 10.3389/fneur.2025.1668912, 41170326 PMC12568028

[17] Gamage PT Dong P Lee J Gharaibeh Y Zimin VN Dallan LAP . Hemodynamic alternations following stent deployment and post-dilation in a heavily calcified coronary artery: in silico and ex-vivo approaches. Comput Biol Med. (2021) 139:104962. doi: 10.1016/j.compbiomed.2021.104962, 34715552 PMC8642291

[18] Malik J Novakova L Valerianova A. Wall shear stress alteration: a local risk factor of atherosclerosis. Curr Atheroscler Rep. (2022) 24:143–51. doi: 10.1007/s11883-022-00993-0, 35080718

[19] Loly VTR Cintra A Ramirez-Velandia F Ogilvy CS Mensah EO de Sá Brasil Lima J . Computational fluid dynamics approaches for analyzing rupture and growth of intracranial aneurysms: a systematic review. Biomedicine. (2025) 13:2914. doi: 10.3390/biomedicines13122914, 41462926 PMC12730923

[20] Li Y Amili O Moen S de Van Moortele PF Grande A Jagadeesan B . Flow residence time in intracranial aneurysms evaluated by in vitro 4D flow MRI. J Biomech. (2022) 141:111211. doi: 10.1016/j.jbiomech.2022.111211, 35780698

[21] Kushner A West WP Khan Suheb MZ Pillarisetty LS. "Virchow triad". In: StatPearls. [Internet]. Treasure Island (FL): StatPearls Publishing (2024) 30969519

[22] Zheng T Tang W Shan Y Guo R Gao Y Tian C . Studying the imaging features and infarction mechanism of vertebrobasilar dolichoectasia with high-resolution magnetic resonance imaging. Brain Pathol. (2023) 33:e13135. doi: 10.1111/bpa.13135, 36718993 PMC10041158

[23] Rzepliński R Tarka S Tomaszewski M Kucewicz M Acewicz A Małachowski J . Narrowings of the deep cerebral perforating arteries ostia: geometry, structure, and clinical implications. J Stroke. (2025) 27:52–64. doi: 10.5853/jos.2024.01655 39916454 PMC11844661

[24] Weiss AJ Panduro AO Schwarz EL Sexton ZA Lan IS Geisbush TR . A matched-pair case control study identifying hemodynamic predictors of cerebral aneurysm growth using computational fluid dynamics. Front Physiol. (2023) 14:1300754. doi: 10.3389/fphys.2023.1300754, 38162830 PMC10757566

[25] Fu Q Ma X Li L Xia W Xie S Biekan J . Decreased wall shear stress on 4D-flow-MRI is associated with wall instability of unruptured intracranial aneurysm. Eur J Radiol. (2025) 190:112200. doi: 10.1016/j.ejrad.2025.112200, 40483771

[26] Wang Y Sun J Li R Liu P Liu X Ji J . Increased aneurysm wall permeability colocalized with low wall shear stress in unruptured saccular intracranial aneurysm. J Neurol. (2021) 269:2715–9. doi: 10.1007/s00415-021-10869-z, 34731309

[27] Morel S Schilling S Diagbouga MR Delucchi M Bochaton-Piallat ML Lemeille S . Effects of low and high Aneurysmal Wall shear stress on endothelial cell behavior: differences and similarities. Front Physiol. (2021) 12:727338. doi: 10.3389/fphys.2021.727338, 34721060 PMC8551710

[28] Zhu Y Zou R Sun X Lei X Xiang J Guo Z . Assessing the risk of intracranial aneurysm rupture using computational fluid dynamics: a pilot study. Front Neurol. (2023) 14:1277278. doi: 10.3389/fneur.2023.1277278, 38187159 PMC10771834

[29] Bozorgpour R. Hemodynamic markers: CFD-based prediction of cerebral aneurysm rupture risk. Vasc Pharmacol. (2026) 162:107578. doi: 10.1016/j.vph.2025.107578, 41455581

[30] Tang Y Wei H Zhang Z Fu M Feng J Li Z . Transition of intracranial aneurysmal wall enhancement from high to low wall shear stress mediation with size increase: A hemodynamic study based on 7T magnetic resonance imaging. Heliyon. (2024) 10:e30006. doi: 10.1016/j.heliyon.2024.e30006, 38694075 PMC11061692

[31] Nagy J Fenz W Miron VM Thumfart S Maier J Major Z . Fluid-structure interaction simulations of the initiation process of cerebral aneurysms. Brain Sci. (2024) 14:977. doi: 10.3390/brainsci14100977, 39451990 PMC11506655

[32] Ramirez-Velandia F de Pimenta Figueiredo VL Loly VTR Koc NA Chieregatti BG Tatit RT . Hemodynamic changes during simulation of sub-maximal handgrip maneuver in small basilar tip aneurysms: a computational fluid dynamics and one-way fluid-structure interaction analysis. Clin Neurol Neurosurg. (2026) 262:109304. doi: 10.1016/j.clineuro.2025.109304, 41478052

[33] Peng C He W Huang X Ma J Yuan T Shi Y . The study on the impact of AAA wall motion on the hemodynamics based on 4D CT image data. Front Bioeng Biotechnol. (2023) 11:1103905. doi: 10.3389/fbioe.2023.1103905, 37064230 PMC10098133

[34] Stahl J McGuire LS Rizko M Saalfeld S Berg P Alaraj A. Are hemodynamics responsible for inflammatory changes in venous vessel walls? A quantitative study of wall-enhancing intracranial arteriovenous malformation draining veins. J Neurosurg. (2024) 141:333–42. doi: 10.3171/2024.1.JNS232625, 38552234

[35] Bouillot P Brina O Ouared R Lovblad KO Farhat M Pereira VM. Particle imaging velocimetry evaluation of intracranial stents in sidewall aneurysm: hemodynamic transition related to the stent design. PLoS One. (2014) 9:e113762. doi: 10.1371/journal.pone.0113762, 25470724 PMC4254651

[36] Korte J Gaidzik F Spitz L Pravdivtseva MS Behme D Larsen N . Analysis of the treatment effect of the contour neurovascular system in intracranial aneurysms: larger neck coverage area is associated with longitudinal flow reduction. Comput Biol Med. (2025) 197:111002. doi: 10.1016/j.compbiomed.2025.111002, 40882484

[37] Chua MMJ Silveira L Moore J Pereira VM Thomas AJ Dmytriw AA. Flow diversion for treatment of intracranial aneurysms: mechanism and implications. Ann Neurol. (2019) 85:793–800. doi: 10.1002/ana.25484 30973965

[38] Wu X Tian Z Liu J Li W Chen J Zhou Y . Hemodynamic impacts of flow diverter devices on the ophthalmic artery. J Transl Med. (2019) 17:160. doi: 10.1186/s12967-019-1913-4, 31096981 PMC6524319

[39] Iantorno M Lipinski MJ Garcia-Garcia HM Forrestal BJ Rogers T Gajanana D . Meta-analysis of the impact of strut thickness on outcomes in patients with drug-eluting stents in a coronary artery. Am J Cardiol. (2018) 122:1652–60. doi: 10.1016/j.amjcard.2018.07.040, 30292330

[40] Siddiqui AH Monteiro A Hanel RA Kan P Mohanty A Cortez GM . Triple therapy versus dual-antiplatelet therapy for dolichoectatic vertebrobasilar fusiform aneurysms treated with flow diverters. J Neurointerv Surg. (2022) 15:655–63. doi: 10.1136/jnis-2022-019151, 36190965

