# Supplementary material for: Perforator-sparing basilar artery reconstruction normalizes pathological hemodynamics in vertebrobasilar dolichoectasia: a CFD study
Source: Front Neurol. 2026 Apr 10;17:1790419. doi: 10.3389/fneur.2026.1790419 (PMC13106077; doi:10.3389/fneur.2026.1790419)
Supplement: Supplementary file 1 [file Table_1.docx]

**Supplementary Table 1. Baseline characteristics and BA morphology of patients**

| **Demographic** | **Group** | | | **P** |
| --- | --- | --- | --- | --- |
|  | **Control** | **Pre** | **Post** |  |
| Women, n (%) | 7 (29.17) | 7 (29.17) | - | - |
| Age | 61.38±8.73 | 61.25±9.27 | - | 0.960 |
| **BA Morphology** | | | |  |
| Volume (mm^3) | 250.07±67.01 | 3645.23±3476.59 | 837.54±506.06 | **< 0.001** |
| Length (mm) | 28.14±3.54 | 52.54±11.76 | - | **< 0.001** |
| Radius (mm) | 1.68±0.24 | 4.21±1.63 | 2.23±0.49 | **< 0.001** |
| Mean area (mm^2) | 9.01±2.93 | 64.17±54.43 | 16.39±6.87 | **< 0.001** |
| Tortuosity | 0.07±0.04 | 0.37±0.28 | - | **< 0.001** |

The bolded values indicate significant differences.
