# Supplementary figures and images for: Perforator-sparing basilar artery reconstruction normalizes pathological hemodynamics in vertebrobasilar dolichoectasia: a CFD study
Source: Front Neurol. 2026 Apr 10;17:1790419. doi: 10.3389/fneur.2026.1790419 (PMC13106077; doi:10.3389/fneur.2026.1790419)

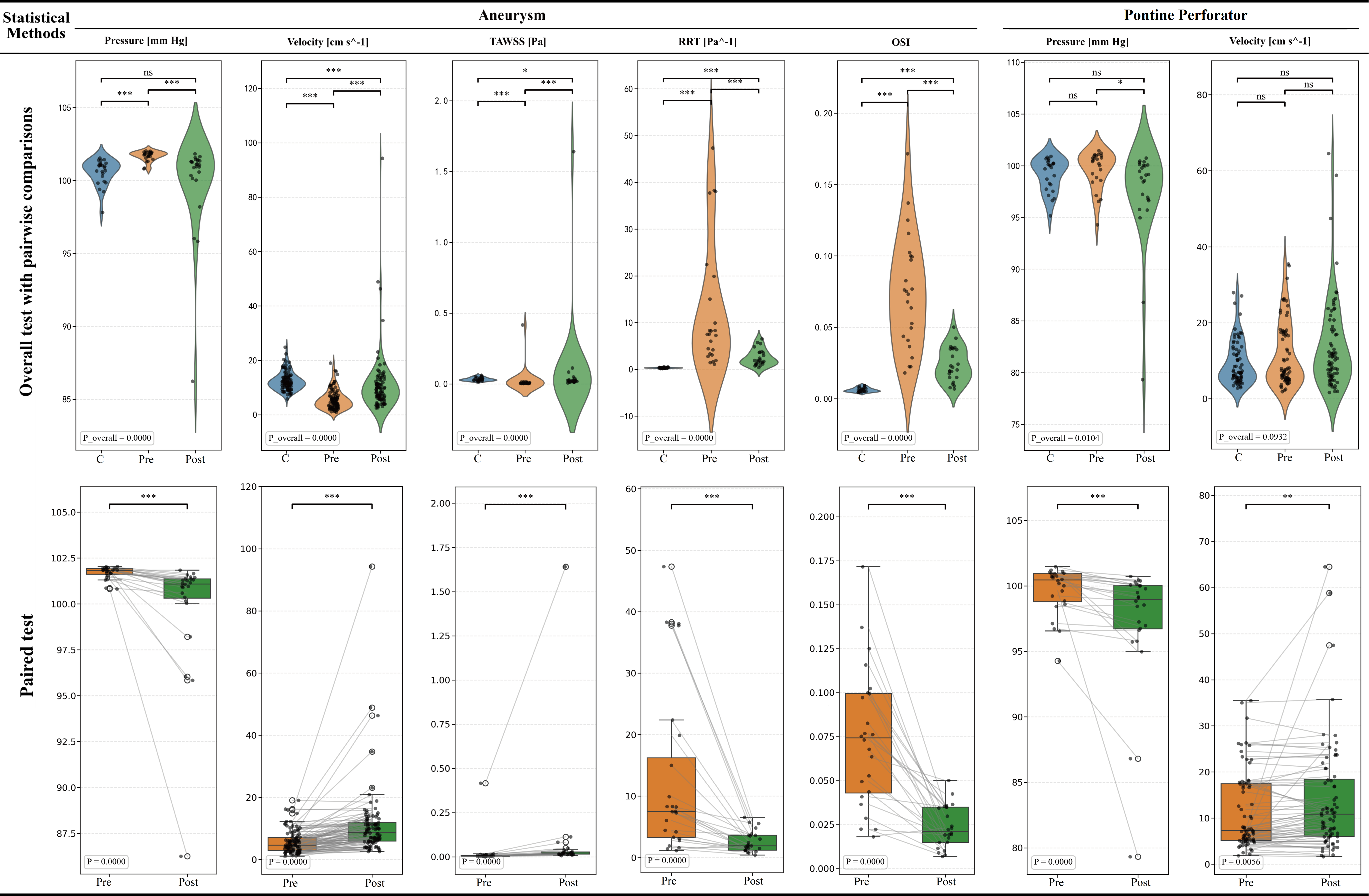

Supplement: Supplementary file 3 [file image_1.png]

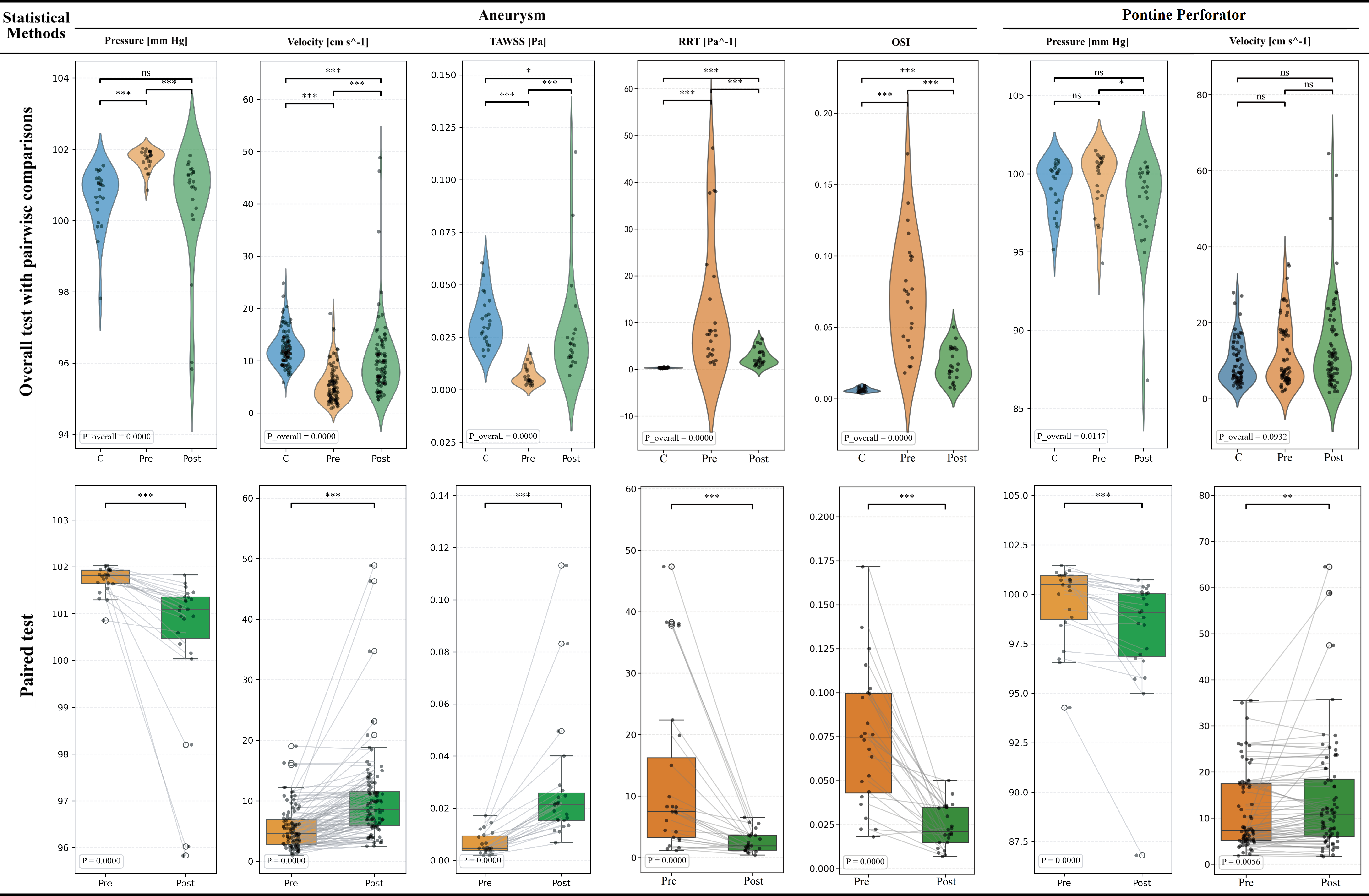

Supplement: Supplementary file 4 [file image_2.png]
